# Unraveling hypoglycemia risk during hemodialysis: a predictive model from a nested case-control study

**DOI:** 10.3389/fphys.2025.1660936

**Published:** 2025-11-10

**Authors:** Jiao Sun, Mohan Ran, Shiying Lv, Jiacheng Li, Hongjing Zan, Wei Li, Qingchu Li

**Affiliations:** 1 The First Clinical Medical College, Shandong University of Traditional Chinese Medicine, Jinan, Shandong, China; 2 Department of Nephrology, The Third Hospital of Shandong Province, Jinan, Shandong, China; 3 Department of Epidemiology and Biostatistics, Institute of Basic Medical Sciences, Chinese Academy of Medical Sciences, School of Basic Medicine, Peking Union Medical College, Beijing, China; 4 School of Public Health, Cheelo College of Medicine, Shandong University, Jinan, Shandong, China; 5 Department of Thoracic Surgery, Qilu Hospital of Shandong University, Jinan, Shandong, China; 6 Department of Nephrology, Affiliated Hospital of Shandong University of Traditional Chinese Medicine, Jinan, Shandong, China

**Keywords:** hemodialysis, hypoglycemia, risk factors, cardiovascular disease, albumin, blood glucose, internal validation, cross-validation

## Abstract

**Background:**

Hemodialysis (HD) can significantly lower blood glucose levels, increasing the risk of hypoglycemia. The contributing factors are not fully understood. This study aimed to identify key risk factors for hypoglycemia during HD and develop a predictive model.

**Methods:**

A retrospective nested case-control study was conducted at the Third Hospital of Shandong Province from January 2020 to December 2023. Clinical and laboratory data were collected from electronic medical records and patient questionnaires. Univariate and multivariate analyses identified independent risk factors, and a predictive model was developed using stepwise logistic regression. Internal validation was performed using 10-fold stratified cross-validation, with model performance evaluated by mean area under the receiver operating characteristic curve (AUC), accuracy, sensitivity, and specificity.

**Results:**

Among 114 HD patients (57 cases, 57 controls), six independent risk factors were identified: afternoon HD session, presence of cardiovascular disease, and low levels of albumin (<37.35 g/L), creatinine (<828.65 μmol/L), urea (<28.05 mmol/L), and pre-dialysis blood glucose (<5.75 mmol/L). The predictive model demonstrated good internal validity with mean AUC 0.79, accuracy 0.71, sensitivity 0.64, and specificity 0.78, indicating stable discriminative performance.

**Conclusion:**

Six key risk factors for hypoglycemia during HD were identified, and a predictive model integrating disease status, HD timing, and laboratory markers was developed. Early identification of high-risk patients may help prevent hypoglycemic events and improve HD outcomes. Future studies should externally validate and refine this model for broader clinical application.

## Introduction

### Background

Kidney failure, resulting from conditions such as chronic kidney disease (CKD) and end-stage renal disease (ESRD), is a growing global public health challenge with significant economic and healthcare burdens (Levey, 2012; KDIGO, 2024). By 2030, approximately 14.5 million people are projected to have ESRD (Bello et al., 2021), and CKD is expected to be the fifth leading cause of death worldwide by 2040 (Foreman et al., 2018). Kidney replacement therapy (KRT) is essential for prolonging survival in patients with kidney failure, with dialysis accounting for 78% of KRT, of which hemodialysis (HD) represents the predominant modality (Pecoits-Filho et al., 2011; Himmelfarb et al., 2020).

Hypoglycemia is a frequent and potentially life-threatening complication in HD patients (Copur et al., 2021). Blood glucose levels fluctuate significantly during HD, particularly within the first 2 h of treatment (Li et al., 2022). Studies have reported lower blood glucose levels on dialysis days compared to non-dialysis days in maintenance HD patients (Kazempour-Ardebili et al., 2009). Hypoglycemia can lead to severe outcomes such as arrhythmias, sudden cardiac death, and stroke (Kofod et al., 2023; Abe and Kalantar-Zadeh, 2015). In diabetic patients, recurrent hypoglycemia can blunt neurohumoral responses, reducing the warning signs of hypoglycemia (Amiel et al., 1988). Even in non-diabetic individuals, a single hypoglycemic episode can impair neuroendocrine function (Heller and Cryer, 1991). The subtle and often unnoticed signs of hypoglycemia make glucose management challenging in HD patients.

Despite the clinical significance of hypoglycemia, its prevention in HD patients remains suboptimal. Studies estimate that the incidence of hypoglycemia during HD in patients with diabetic nephropathy ranges from 23.8% to 47.6% (Ricks et al., 2012; Chu et al., 2017). Although continuous glucose monitoring has been suggested as a potential solution (Galindo and Aleppo, 2020), its clinical adoption is limited due to cost and complexity (Galindo et al., 2022). Given these challenges, identifying risk factors for hypoglycemia and developing predictive strategies are crucial for improving patient outcomes. CGM provides real-time glucose monitoring during HD, allowing timely interventions such as dietary supplementation, insulin titration, or adjustment of dialysis prescriptions to counteract hypoglycemia.

### Rationale for study design

A nested case–control study is an epidemiological approach that combines the strengths of cohort and case–control designs, effectively minimizing selection bias by selecting both case and control subjects from the same well-defined source population (Dey et al., 2020). This method ensures temporal comparability between cases and controls and allows efficient utilization of existing retrospective data.

In the present study, the nested case–control design was chosen not because hypoglycemia during hemodialysis is rare, but because it enables efficient analysis within a large hemodialysis cohort while preserving the temporal sequence between clinical exposures and hypoglycemic events. This approach reduces data extraction burden, facilitates inclusion of multiple clinical and treatment-related variables, and enhances internal validity by selecting controls from the same risk set as the cases. Nested case–control designs have been successfully applied in various medical investigations, such as studies on traveler’s diarrhea (Schaumburg et al., 2020), the association between plasma trimethylamine N-oxide (TMAO) and stroke risk (Liu et al., 2023), and drug safety evaluations (Daneman et al., 2021).

### Knowledge gaps and study objectives

Despite the high incidence of hypoglycemia in HD patients, few studies have comprehensively investigated its risk factors, particularly those occurring during the HD session. Existing studies primarily focus on diabetic patients or rely on cross-sectional designs, overlooking key laboratory markers and broader patient populations (Simic-Ogrizovic et al., 2001; Kang et al., 2023). While previous research has linked hypoglycemia to factors such as age, gender, race, dialysis session length, and residual renal function (Kang et al., 2023), the role of laboratory-based parameters remains underexplored. Additionally, metabolic changes during HD, including altered gluconeogenesis, reduced insulin clearance, glucose loss to the dialysate, and intracellular glucose diffusion, contribute to hypoglycemia but are difficult to monitor in routine clinical practice (Abe and Kalantar-Zadeh, 2015). Recent efforts to develop predictive models have been limited to diabetic nephropathy patients, restricting their broader applicability (Zhang et al., 2023).

To address these gaps, we conducted a nested case-control study to identify comprehensive risk factors for hypoglycemia during HD and develop a predictive model. Unlike prior studies, we examined the entire HD patient population, including both diabetic and non-diabetic individuals, with a particular focus on hypoglycemic events occurring during HD. Furthermore, to enhance the scientific rigor and ensure model reliability, we incorporated an internal validation step using 10-fold stratified cross-validation. This approach allowed us to evaluate the predictive model’s stability and generalizability within the study cohort, thereby reducing the potential for overfitting. Our findings aim to provide a scientific foundation for early risk stratification and targeted preventive strategies to reduce hypoglycemia-related complications in HD patients. While previous studies, including a recent investigation using CGM in both diabetic and non-diabetic patients (Piersanti et al., 2025), have also explored hypoglycemia during HD, our study differs in its use of stepwise logistic regression to build a risk-prediction model based on routinely available clinical and laboratory parameters and in performing internal validation to confirm the robustness of the predictive model.

## Materials and methods

### Study design and setting

This study was a retrospective nested case-control study conducted at the Department of Nephrology, The Third Hospital of Shandong Province from 1 January 2020, to 31 December 2023. A total of 114 patients undergoing maintenance hemodialysis (HD) were enrolled. The case group consisted of 57 patients who experienced hypoglycemia during HD, while the control group included 57 patients who did not develop hypoglycemia. Controls were randomly selected from the same HD cohort at the time each case was identified to ensure comparability. Kidney transplant recipients who returned to HD were not included in this study. The etiology of kidney failure leading to HD (e.g., diabetic nephropathy, glomerulonephritis, polycystic kidney disease) was not systematically analyzed in this dataset, representing a potential limitation.

### Definition of hypoglycemia during HD

Hypoglycemia during HD was defined as a blood glucose (GLU) level below 70 mg/dL (3.9 mmol/L), with or without clinical symptoms, occurring at least once during an HD session. Blood glucose measurements were obtained using Sinocare 1,000 glucometers (glucose dehydrogenase method) at standardized points during each HD session, including pre-dialysis, hourly during dialysis, and post-dialysis.

### Eligibility criteria

Patients were eligible for inclusion if they were ≥18 years of age, stable outpatients who had been receiving HD for at least 3 months, and were undergoing HD three times per week for 4 h per session. The case group comprised patients who experienced at least one episode of hypoglycemia during HD, while the control group included those who had never experienced hypoglycemia during HD.

Patients were excluded if they were lost to follow-up during the study period, had missing key demographic or clinical data (such as gender, age, height, or weight), had severe comorbidities requiring intensive care (e.g., ICU admission), or were unable to complete the questionnaire due to cognitive or physical limitations.

### Data collection

A structured one-on-one questionnaire survey was conducted by a trained researcher to collect data on demographic characteristics, disease history, and HD conditions. Written informed consent was obtained from all participants before data collection. Additionally, laboratory test results were extracted from the hospital’s electronic medical records (EMR) to ensure comprehensive data collection. The parameters assessed included mean arterial pressure (MAP), C-reactive protein (CRP), hemoglobin (HGB), red blood cell count (RBC), albumin (ALB), prealbumin (PAB), triglycerides (TG), total cholesterol (TC), creatinine (Cre), urea, and blood glucose (GLU). These laboratory values were carefully verified for accuracy to maintain the reliability of the study findings.

All laboratory parameters were obtained from routine pre-dialysis investigations conducted within 1 week prior to the index hemodialysis session. These pre-HD values were considered baseline biochemical data for both case and control participants to ensure consistency and comparability.

### Statistical analysis

Data analyses were performed using IBM SPSS Statistics 26, and graphical visualizations were generated with GraphPad Prism 10.1.2. The Kolmogorov-Smirnov test assessed normality. Descriptive statistics summarized data distributions: normally distributed variables were expressed as mean ± standard deviation (SD) and compared using the t-test, while non-normally distributed variables were presented as median (interquartile range, IQR) and analyzed with the Mann-Whitney U test. Categorical variables were expressed as counts (percentages) and compared using the Chi-square test.

Variables with p < 0.1 in univariate analysis were included in a multiple stepwise logistic regression model to identify independent risk factors for hypoglycemia during HD. Multicollinearity among predictors was assessed using the variance inflation factor (VIF). Cut-off values were established for continuous variables to enhance interpretability, followed by subgroup analyses. Model performance was evaluated using the Hosmer-Lemeshow goodness-of-fit test and the receiver operating characteristic (ROC) curve. A p-value <0.05 was considered statistically significant.

To further assess the internal robustness of the predictive model, a 10-fold cross-validation procedure was performed. The dataset was randomly partitioned into ten equal subsets. In each iteration, nine subsets were used for model training and one subset for validation. This process was repeated ten times so that each subset served once as a validation set. The average performance across all folds was used to estimate the stability and generalizability of the model within the study population. A p-value <0.05 was considered statistically significant.

## Results

### Baseline characteristics

**FIGURE 1:**
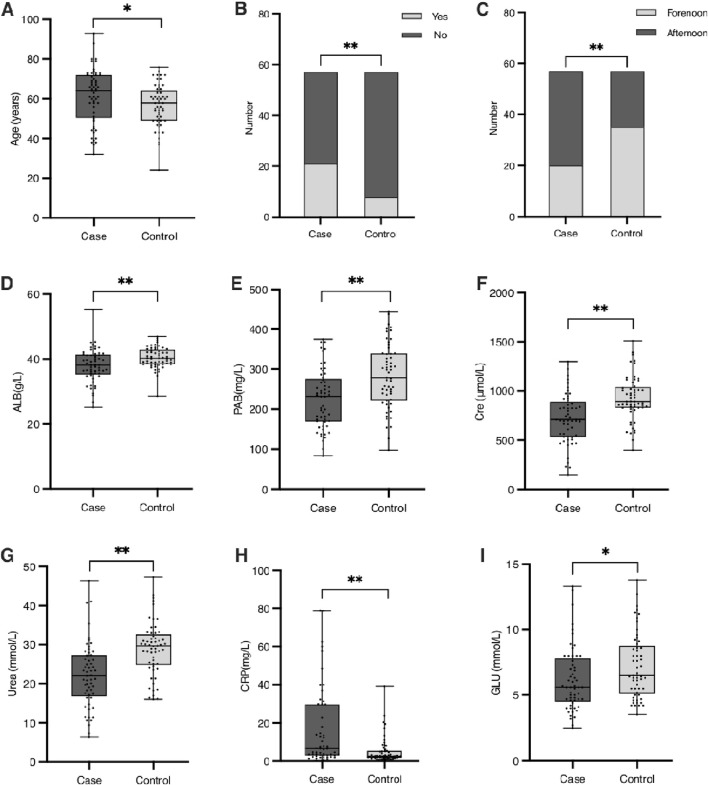
Baseline characteristics of patients with significant differences between the case and control groups. **(A)** Age (years). **(B)** Number of patients with or without cardiovascular diseases (CVDs) in the case and control groups. **(C)** Distribution of HD treatment periods (forenoon vs. afternoon) in the case and control groups. **(D)** Serum albumin concentration (g/L). **(E)** Serum prealbumin concentration (g/L). **(F)** Serum creatinine concentration (μmol/L). **(G)** Serum urea concentration (mmol/L). **(H)** Red blood cell (RBC) count (×10^12^/L). **(I)** Blood glucose levels (mmol/L). Significant differences between groups are indicated as *(p < 0.05) or **(p < 0.01).

A total of 114 patients undergoing maintenance hemodialysis (HD) were included, with 57 patients in the hypoglycemia group and 57 in the control group. Patients in the hypoglycemia group were significantly older than controls (mean ± SD: 65.2 ± 9.1 vs. 59.8 ± 10.3 years, p < 0.05), and the prevalence of cardiovascular diseases (CVDs) was nearly three times higher compared to controls (49.1% vs. 16.7%, p < 0.01). Timing of HD sessions also differed significantly, with a higher proportion of patients undergoing afternoon HD experiencing hypoglycemia, whereas controls were more frequently treated in the forenoon (p < 0.01). Laboratory findings revealed notable differences between the groups: the inflammatory marker C-reactive protein (CRP) was markedly elevated in the hypoglycemia group (median [IQR]: 12.8 [8.4–17.6] mg/L vs. 4.3 [2.1–7.9] mg/L, p < 0.01), while nutritional markers, including albumin (ALB) and prealbumin (PAB), were significantly lower (ALB: 34.5 ± 4.2 g/L vs. 39.1 ± 3.8 g/L, p < 0.01; PAB: 0.20 ± 0.07 g/L vs. 0.28 ± 0.08 g/L, p < 0.01). Kidney function markers also differed, with lower creatinine (689 ± 142 μmol/L vs. 889 ± 135 μmol/L, p < 0.01) and urea levels (24.2 ± 5.6 mmol/L vs. 31.2 ± 6.3 mmol/L, p < 0.01) in the hypoglycemia group. Blood glucose (GLU) levels were lower in the hypoglycemia group (5.32 ± 0.42 mmol/L vs. 6.31 ± 0.49 mmol/L, p < 0.05). A detailed comparison is presented in Table 1 and Figure 1. Other factors—including gender, body mass index (BMI), diabetes, hypertension, high nutritional risk, food intake during HD, glucose injection during HD, mean arterial pressure (MAP), hemoglobin (HGB), red blood cell count (RBC), triglycerides (TG), and total cholesterol (TC)—were not significantly associated with hypoglycemia. Despite previous literature suggesting potential influences of gender and nutritional risk, our findings did not support these associations.

**TABLE 1:** Baseline patient characteristics.

Variable	Total (*n* = 114)	Control (*n* = 57)	Case (*n* = 57)
Male	69 (60.5%)	39 (68.4%)	30 (52.6%)
Age (years)	59.05 ± 12.47	56.61 ± 10.50	61.49 ± 13.84*
BMI (kg/m^2^)	22.74 ± 3.23	22.40 ± 3.23	23.09 ± 3.21
Severe nutritional risk	12 (10.5%)	5 (8.8%)	7 (12.3%)
Hypoglycemia during HD interval	46 (40.4%)	21 (36.8%)	25 (43.9%)
Diabetes	52 (45.6%)	25 (43.9%)	27 (47.4%)
Hypertension	100 (87.7%)	50 (87.7%)	50 (87.7%)
CVDs	29 (25.4%)	8 (14.0%)	21 (36.8%)**
HD period – Forenoon	55 (48.2%)	35 (61.4%)	20 (35.1%)**
HD period – Afternoon	59 (51.8%)	22 (38.6%)	37 (64.9%)**
Food intake during HD	59 (51.8%)	25 (43.9%)	34 (59.6%)
Glucose injection during HD	15 (13.2%)	5 (8.8%)	10 (17.5%)
MAP (mmHg)	105.53 ± 12.67	106.40 ± 12.76	104.66 ± 12.62
CRP (mg/L), M (IQR)	3.50 (10.725)	2.50 (4.10)	6.40 (26.85)**
HGB (g/L)	108.17 ± 16.85	110.31 ± 11.81	106.03 ± 20.59
RBC (10^12^/L)	3.50 ± 0.55	3.50 ± 0.44	3.49 ± 0.65
ALB (g/L)	39.14 ± 4.47	40.40 ± 3.20	37.89 ± 5.18**
PAB (mg/L)	254.05 ± 81.97	282.79 ± 80.34	225.31 ± 3.64**
TG (mmol/L)	1.28 ± 0.81	1.14 ± 0.65	1.34 ± 1.07
TC (mmol/L), M (IQR)	3.52 (1.01)	3.43 (0.85)	3.58 (1.14)
Cre (μmol/L)	823.93 ± 263.00	924.81 ± 226.24	723.05 ± 260.15**
Urea (mmol/L)	25.56 ± 8.34	28.99 ± 6.90	22.13 ± 8.28**
GLU (mmol/L)	6.15 ± 3.30	6.50 ± 3.65	5.60 ± 3.30*

HD, hemodialysis; severe nutritional risk was evaluated using Nutritional Risk Screening (NRS, 2002), where the total NRS, score is the sum of the nutrition score, disease severity score, and age adjustment. Severe risk was defined as NRS ≥3. Hypoglycemia during the HD, interval was defined as blood glucose <70 mg/dL measured between hemodialysis sessions, including both control and case groups. Variables from MAP, to GLU, were all measured before HD., Values are presented as mean ± SD, median (interquartile range), or n (%). *p < 0.05 vs. control; **p < 0.01 vs. control.

### Multiple stepwise logistic regression analysis

**FIGURE 2:**
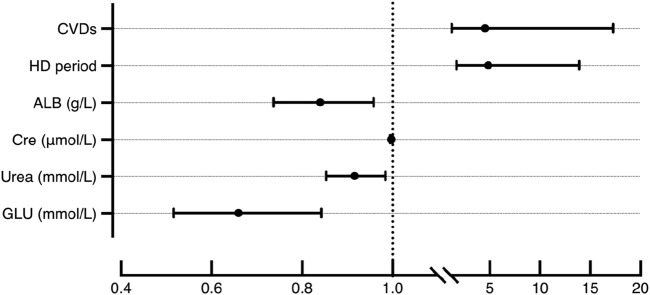
Odds ratio (95%CI) of CVDs, HD period, ALB, Cre, Urea and GLU for hypoglycemia during HD.

Stepwise logistic regression identified several independent determinants of hypoglycemia during hemodialysis (HD) (Table 2) (Figure 2). Cardiovascular diseases (CVDs) significantly increased the risk of hypoglycemia, with an odds ratio (OR) of 4.54 (95% CI: 1.70–12.11, p < 0.05). Timing of HD sessions was also an independent predictor, with patients undergoing afternoon HD having a higher risk compared to those treated in the forenoon (OR = 4.85, 95% CI: 1.85–12.70, p < 0.05). Therefore, it is advisable to avoid receiving HD too late in the day to better prevent the occurrence of hypoglycemia, especially for patients with severe glycemic regulation impairments.

**TABLE 2:** Multiple stepwise logistic regression analysis of hypoglycemia during HD.

Variable	β	S	Wald χ^2^	OR (95% CI)	p
CVDs	1.51	0.68	4.93	4.54 (1.19, 17.28)	0.027*
HD period	1.58	0.54	8.65		
Forenoon				1.00	
Afternoon				4.85 (1.69, 13.91)	0.003**
ALB (g/L)	−0.17	0.07	6.78	0.84 (0.74, 0.96)	0.009**
Cre (μmol/L)	−0.003	0.001	4.35	0.99 (0.99, 1.00)	0.037*
Urea (mmol/L)	−0.09	0.04	5.84	0.926 (0.85, 0.98)	0.016*
GLU (mmol/L)	−0.42	0.13	11.033	0.66 (0.52, 0.84)	0.001*

β, regression coefficient; S standard error; OR, odds ratio; CI, confidence interval.

Biochemical factors demonstrated strong associations with hypoglycemia risk. Albumin (ALB) levels were inversely related to hypoglycemia occurrence (OR = 0.84, 95% CI: 0.71–0.98, p < 0.05), and lower pre-HD glucose (GLU) was similarly associated with greater hypoglycemia risk (OR = 0.66, 95% CI: 0.44–0.97, p < 0.05). Interestingly, higher creatinine (Cre) (OR = 1.00, 95% CI: 0.99–1.00, p < 0.05) and urea (OR = 0.92, 95% CI: 0.85–0.99, p < 0.05) appeared to have a protective effect. Variables such as age, C-reactive protein (CRP), and prealbumin (PAB) were excluded from the final model. Although age differed significantly between groups in univariate analysis, it was not retained in the multivariable model, likely due to collinearity with cardiovascular diseases and renal function parameters, which accounted for a larger portion of the variance in hypoglycemia risk. Similarly, PAB was excluded due to its strong correlation with ALB.

**FIGURE 3:**
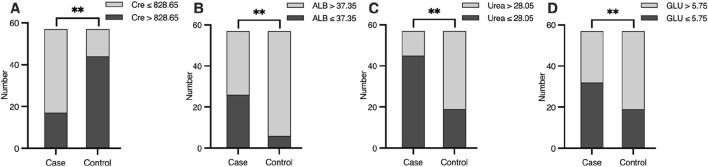
Differences of **(A)** ALB (g/L), **(B)** Cre (μmol/L), **(C)** Urea (mmol/L) and **(D)** GLU (mmol/L) level between case and control group, according to the cut-off value. Significant differences are marked as *(*P* < 0.05) or **(*P* < 0.01).

To refine the predictive model, receiver operating characteristic (ROC) curve analysis was used to determine optimal cut-off values for continuous predictors (Table 3). For ALB, a threshold of 37.35 g/L identified 45.6% of hypoglycemia patients below this value compared to 10.5% of controls (p < 0.01, Figure 3A). For Cre, a cut-off of 828.65 μmol/L captured 70.2% of hypoglycemia patients versus 22.8% of controls (p < 0.01, Figure 3B). For urea, a threshold of 28.05 mmol/L included 78.9% of hypoglycemia patients below this level compared to 33.3% of controls (p < 0.01, Figure 3C). Finally, for GLU, a cut-off of 5.75 mmol/L identified 56.1% of hypoglycemia patients versus 33.3% of controls (p < 0.01, Figure 3D). These thresholds provide practical clinical indicators for identifying HD patients at greater risk of hypoglycemia.

**TABLE 3:** In-group analysis grouped by cut-off value.

Variable	Case	Control	*χ2*	p
ALB (g/L)				
≤37.35	26	6	17.378	<0.001
>37.35	31	51		
Cre (μmol/L)				
≤828.65	40	13	25.706	<0.001
>828.65	17	44		
Urea (mmol/L)				
≤28.05	45	19	24.082	<0.001
>28.05	12	38		
GLU (mmol/L)				
≤5.75	32	19	5.996	0.014
>5.75	25	38		

Sensitivity and specificity in Table 3 are based on raw subgroup counts, whereas Table 4 presents ROC-derived sensitivity and specificity at the optimal cut-off values (Youden’s index). Differences between the tables do not indicate errors.

**TABLE 4:** ROC curve analysis of ALB, Cre, Urea, Glucose on hypoglycemia during HD.

Variable	AUC	Cut-off value	Sensitivity (%)	Specificity (%)	Youden’s index	p
ALB (g/L)	0.671	37.35	89.5	45.6	0.351	0.001
Cre (μmol/L)	0.730	828.65	77.2	70.2	0.474	<0.001
Urea (mmol/L)	0.758	28.05	66.7	78.9	0.456	<0.001
GLU (mmol/L)	0.629	5.75	66.7	56.1	0.228	0.014

AUC, area under the curve. ROC-derived sensitivity/specificity may differ from raw counts in Table 3 due to the method of determining the optimal cut-off (Youden’s index).

### Construction and evaluation of prediction model

A logistic prediction model was constructed based on the six independent determinants identified in the multiple stepwise logistic regression analysis. The formula for the model is:

$$P=1/\left[1+e\left(13.083+1.512^{*}\text{ CVDs}+1.580^{*}\text{ HD period}‐0.174^{*}\text{ ALB}‐0.003^{*}\text{ Cre}‐0.087^{*}\text{ Urea}‐0.416^{*}\text{ GLU}\right)\right]$$

where P represents the probability of experiencing hypoglycemia during HD. The closer P is to 1, the higher the likelihood of hypoglycemia occurrence, whereas a value closer to 0 suggests a lower risk.

**FIGURE 4:**
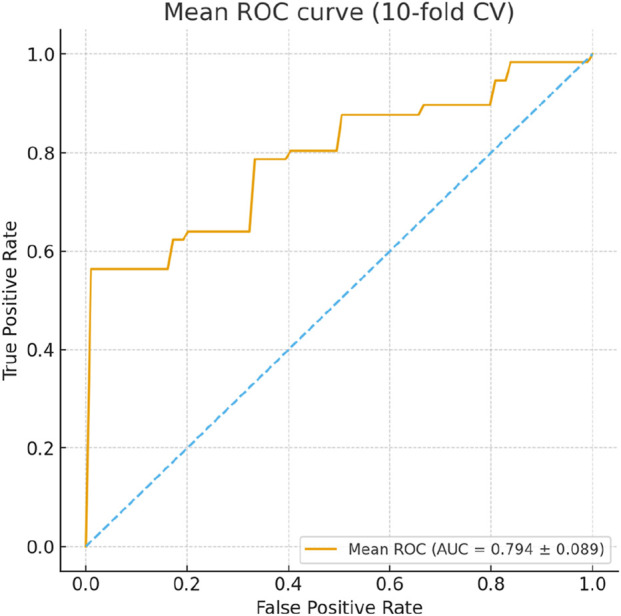
ROC curves from 10-fold cross-validation of the logistic regression model predicting hypoglycemia during hemodialysis. The mean AUC was 0.794 (SD = 0.165), with accuracy 0.737 (SD = 0.100), sensitivity 0.759 (SD = 0.160), and specificity 0.666 (SD = 0.173), demonstrating consistent internal performance across folds.

**FIGURE 5:**
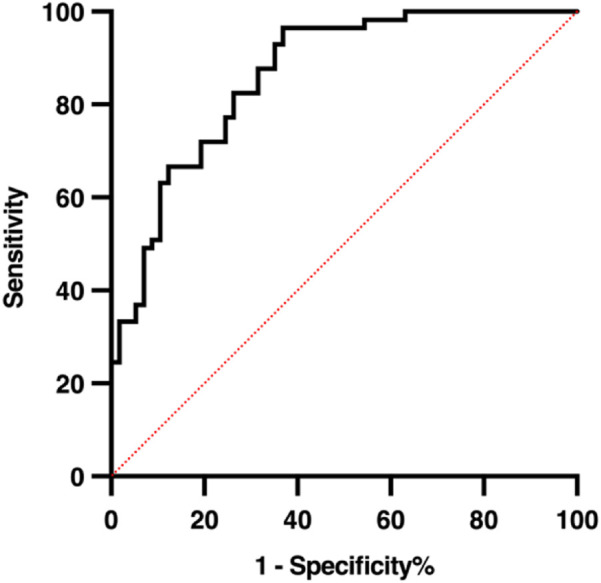
The ROC curve of the prediction model.

To assess the reliability and predictive performance of the model, the Hosmer-Lemeshow goodness-of-fit test was conducted, yielding a value of 12.275 (p = 0.139), indicating no significant difference between the predicted and observed outcomes and suggesting a good model fit. Additionally, the area under the receiver operating characteristic (ROC) curve (AUC) was 0.864 (95% CI: 0.798–0.929, p < 0.001), demonstrating excellent discriminative ability. In the 10-fold cross-validation, the model demonstrated consistent discriminative ability with a mean AUC of 0.794 (SD = 0.165), accuracy of 0.737 (SD = 0.100), sensitivity of 0.759 (SD = 0.160), and specificity of 0.666 (SD = 0.173) across folds (Figure 4). These results indicate reliable internal performance, suggesting that the model generalizes well within the available cohort. These results suggest that the logistic prediction model shows potential for estimating the likelihood of hypoglycemia in HD patients and could support early intervention and risk management; however, external validation is needed before its clinical application can be confirmed. A graphical representation of the prediction model is shown in Figure 5.

## Discussion

The occurrence of hypoglycemia is not uncommon in HD patients, indicating a potential danger that should not be overlooked. Therefore, this study identified several risk factors and constructed a prediction model to help reduce the likelihood of hypoglycemia during HD. According to our study, patients who received HD in the afternoon were more likely to experience hypoglycemia episodes than those who had treatments in the forenoon. A study in 2021 also noted that the timing of HD is associated with HD-related and post-HD hypoglycemia (Hayashi et al., 2021). In HD patients, insulin-mediated stimulation of peripheral glucose disposal by muscle and adipose tissue is significantly affected, while hepatic glucose uptake continues normally, and hepatic glucose production can be suppressed (Williams and Garg, 2014). Additionally, human muscle tissue exhibits a diurnal rhythm in insulin sensitivity and mitochondrial oxidative capacity (Stenvers et al., 2019). Recent medicinal chemistry studies have further provided molecular insights into glucose-lowering mechanisms of compounds such as pyrazoline and 2,4-thiazolidinedione derivatives, which are relevant for glycemic regulation in susceptible patients (Sharma et al., 2023; Malik and Singh, 2025). These factors may explain the differences in hypoglycemia incidence related to the timing of HD treatments. Therefore, it is advisable to avoid receiving HD too late in the day to better prevent the occurrence of hypoglycemia, especially for those with severe glycemic regulation impairments.

HD patients with CVDs had a higher incidence of hypoglycemia during HD in our study. Previous studies have suggested that hypoglycemia may exacerbate CVDs (Goto et al., 2016). During acute hypoglycemia, heart rate and systolic blood pressure increase, blood flow in the myocardium increases, and cardiac output, stroke volume, and myocardial contractility also rise (Snell-Bergeon and Wadwa, 2012). These changes can place substantial stress on the cardiovascular system. Given the high prevalence of CVDs among patients who experienced hypoglycemia during HD in our study, it may imply that hypoglycemia in HD patients with CVDs could lead to repeated aggravation of CVDs. In this way, hypoglycemia and CVDs can promote each other and form a vicious cycle. We plan to continue focusing on the interaction mechanisms and clinical trends between CVDs and hypoglycemia in the near future. Preventing hypoglycemia during HD might be a critical step in breaking this cycle and potentially improving the prognosis for HD patients with CVDs.

We observed that the incidence rate of hypoglycemia during HD increased with the severity of ALB deficiency. In our study, HD patients with ALB levels below 37.35 g/L were particularly vulnerable to hypoglycemia during HD, according to the cut-off value analysis result. This cut-off value aligns closely with findings from an earlier study in Japan on diabetics treated with insulin (Kawaguchi et al., 2019). Furthermore, increased malnutrition risk has been associated with hypoglycemia occurrence in some studies. ALB, as an indicator of nutritional status, is closely related to human nutrition levels (Ricks et al., 2012; Leibovitz et al., 2018). Since HD is not only a method of elimination but also a process of consumption, it results in significant nutrient loss, leading to malnutrition in HD patients (Kalantar-Zadeh et al., 2004). For instance, there may be inevitable loss of amino acids and albumin into the dialysate, and the inflammatory stimuli associated with the dialysis procedure can lead to increased protein metabolism (Ikizler et al., 2013). Therefore, achieving and maintaining a relatively high nutritional status and ALB level is crucial for HD patients. It is important to focus on a reasonable diet and adequate nutrient intake to achieve optimal nutrition goals, including maintaining ALB levels.

Our study found that HD patients with relatively low Cre and urea levels were prone to hypoglycemia during HD, suggesting that greater adequacy of HD was more likely to trigger hypoglycemia. Normally, metabolic waste in the human body, including Cre and urea, should be sufficiently eliminated after effective HD. The lower the Cre and urea levels, the more adequate the HD treatment received. However, given the high incidence rate of hypoglycemia during HD as per our results, the adequacy of the HD therapy should be reconsidered. Since all patients in our study were treated with the same HD therapy schedule and the same HD fluid, it could be implied that the high clearance of Cre and urea by the HD therapy schedule may be accompanied by more clearing of glucose. This could potentially increase the risk of hypoglycemia. Moreover, several studies have linked low serum creatinine to diabetes (Bao et al., 2018; Hu et al., 2019; Hayes and Kriska, 2008), suggesting that low serum creatinine may influence blood glucose conditions. These findings demonstrate a close relationship between the sufficiency of HD and the occurrence of hypoglycemia during HD. Thus, we suggest that individualized therapy with appropriate HD adequacy should be made based on recent Cre and urea levels. However, the optimal HD adequacy for reducing the high risk of hypoglycemia during HD still requires further study.

We also found that patients with lower GLU levels before HD, particularly those below 5.75 mmol/L, were more susceptible to experiencing hypoglycemia episodes during HD. An earlier report indicated that the percentage change in peri-dialytic blood glucose was associated with the percentage change in pre-HD glucose, which corroborates our findings (Bao et al., 2018; Watha et al., 2022). Additionally, previous data have demonstrated that blood glucose fluctuation during HD may be due to blood flows to the liver, muscle, and heart, which ultimately affect tissue glucose uptake (Foss et al., 1996). It is not difficult to speculate that lower GLU levels before HD may increase the risk of hypoglycemia induced by HD. Therefore, we advise against controlling GLU levels at a very low level before HD, although strict blood glucose management is necessary for HD patients. According to our study, the GLU level before HD should not be lower than 5.75 mmol/L to prevent hypoglycemia during HD (Kondrup et al., 2003).

To further assess the robustness and reliability of the prediction model, internal validation was performed using 10-fold cross-validation. The model demonstrated consistent discriminative ability with a mean AUC of 0.794 (SD = 0.165), accuracy of 0.737 (SD = 0.100), sensitivity of 0.759 (SD = 0.160), and specificity of 0.666 (SD = 0.173) across folds (Figure 4). These results indicate reliable internal performance, suggesting that the model generalizes well within the available cohort, supporting its potential clinical utility for early identification of HD patients at risk of hypoglycemia.

### Limitations

This study has several limitations. Firstly, medication use, which may significantly influence hypoglycemia occurrence during HD, was not included in the analysis due to the complexity of assessing various drug effects and limitations in the hospital’s electronic health records, which may have affected the accuracy and applicability of the findings. Secondly, the nested case-control study design inherently has lower statistical power compared to the larger parent cohort, potentially limiting generalizability. Additionally, as a retrospective study with a relatively small sample size, establishing strong causal relationships between risk factors and hypoglycemia was difficult. Thirdly, continuous variables such as albumin, creatinine, urea, and pre-dialysis glucose were dichotomized for clinical interpretability, which may result in some loss of information and reduced statistical power; however, the logistic regression coefficients still reflect their continuous relationships. Fourthly, while the predictive model underwent internal validation using the Hosmer–Lemeshow goodness-of-fit test, ROC curve analysis, and 10-fold cross-validation, external validation or bootstrapping was not performed, which may limit its generalizability. Furthermore, certain factors could not be analyzed due to data limitations, including insulin or hypoglycemic medications, beta-blocker use, dialysate glucose concentration, tissue-level glucose/insulin dynamics, and individualized dialysis planning. Some associations observed, such as lower creatinine and urea appearing protective, are based on observational data and should be interpreted cautiously. The observation window for hypoglycemia was also limited to available medical record data, and the duration of “never experiencing hypoglycemia” may be insufficient to capture all events, potentially introducing selection bias.

These limitations highlight areas for future prospective studies with larger, multicenter cohorts and more comprehensive datasets—including medication history, dietary intake, and dialysis parameters—to confirm and strengthen the robustness, clinical applicability, and biological interpretation of hypoglycemia risk prediction in HD patients.

## Conclusion

In summary, this study highlights HD period, CVDs, ALB, Cre, Urea, and GLU as key factors for preventing hypoglycemia during HD. The incidence of hypoglycemia during HD may be reduced by implementing strategies such as avoiding HD late in the day, promptly managing CVDs, maintaining adequate nutritional status particularly ALB levels, individualizing HD adequacy to prevent excessive clearance, and avoiding lowering blood glucose to very low levels before HD. While these recommendations provide a practical framework, further interventional studies are required to validate and refine these strategies. Clinical trials could help determine optimal HD timing, the most effective management of CVDs, and precise nutritional and glycemic targets to minimize hypoglycemic risk. Additionally, future research may identify other factors contributing to hypoglycemia during HD, expanding the scope of preventive measures. Ongoing evidence-based investigations are crucial to improve the safety and quality of care for HD patients.

## Data Availability

The original contributions presented in the study are included in the article/supplementary material, further inquiries can be directed to the corresponding authors.
